# The epidemiology and survival of extrapulmonary small cell carcinoma in South East England, 1970–2004

**DOI:** 10.1186/1471-2407-9-209

**Published:** 2009-06-29

**Authors:** Yien Ning S Wong, Ruth H Jack, Vivian Mak, Møller Henrik, Elizabeth A Davies

**Affiliations:** 1 King's College London, Thames Cancer Registry, 1st Floor, Capital House, 42 Weston Street, London SE1 3QD, UK

## Abstract

**Background:**

Extrapulmonary small cell carcinoma (EPSCC) is a rare cancer and few studies describe its epidemiology. Our objectives were to compare the incidence and survival of EPSCC in South East England with small cell carcinoma of the lung (SCLC), to determine the most common anatomical presenting sites for EPSCC and to compare survival in EPSCC by disease stage and site of diagnosis.

**Methods:**

We used data from the Thames Cancer Registry database for South East England between 1970 and 2004 to determine the incidence, most common anatomical sites, and survival by site, and stage of EPSCC. 1618 patients registered with EPSCC were identified. We calculated the age-standardised incidence rate for EPSCC using the European standard population and compared this to that for SCLC. We calculated survival using the Kaplan-Meier method for EPSCC and SCLC, and reported 3-year survival for different EPSCC anatomical sites and disease stages.

**Results:**

The incidence of EPSCC was much lower than for SCLC, similar in males and females, and stable throughout the study period, with incidence rates of 0.45 per 100,000 in males and 0.37 in females during 2000–2004. In general, patients with EPSCC had a better 3-year survival (19%) than SCLC (5%). The most common anatomical sites for EPSCC were oesophagus (18%), other gastrointestinal (15%), genitourinary (20%), head and neck (11%), and breast (10%). Breast EPSCC had the best 3-year survival (60%) and gastrointestinal EPSCC the worst (7%).

**Conclusion:**

This study suggests that EPSCC has a stable incidence and confirms that it presents widely, but most commonly in the oesophagus and breast. Site and extent of disease influence survival, with breast EPSCC having the best prognosis. Further studies using standardised diagnosis, prospective case registers for uncommon diseases and European cancer registries are needed to understand this disease.

## Background

Neuroendocrine tumours can be broadly classified into three groups: well differentiated tumours (true carcinoids), moderately differentiated tumours (atypical carcinoids) and poorly differentiated tumours (small cell carcinomas) [1]. The latter group includes extrapulmonary small cell carcinoma (EPSCC) and small cell lung cancer (SCLC). EPSCC is a rare entity, and in the United States it accounts for approximately 2.5 to 5% of all small cell carcinomas [2-4]. The term came into use in the 1990s and various descriptions including "oat cell" and "extrapulmonary oat cell carcinoma" have been used since the 1970s. The first description by Duguid and Kennedy in 1930 was of the disease occurring in the mediastinum [5], and since then EPSCC has been reported to have arisen in virtually every site of the body [3,5-7]. EPSCC often presents with a mixed morphology of small cell carcinoma and various other epithelial cell types. It is now widely accepted that it derives from a pluri-potent stem cell that develops neuroendocrine features [8], rather than the initial speculation of its origin being from the Amine Precursor Uptake and Decarboxylase (APUD) cells. There is also recent molecular evidence that small cell neoplasms may occur as a late-stage phenomenon in genetically associated organ-typical carcinomas [9]. Despite these new findings, EPSCC is still poorly appreciated and clinically it may be confused with metastases from SCLC.

The prognosis of patients with EPSCC is generally unfavourable as is the case for those with SCLC. This is due to an aggressive natural history of EPSCC – a course characterised by rapid local progression, early, widespread metastases and recurrence after treatment [3]. The epidemiological and clinical literature describing EPSCC is relatively limited. One of the most recent and largest studies reported a series of 101 patients and found an overall median survival of 9.83 months after diagnosis [6].

Although there are a number of single institution studies of EPSCC [10,11] we are not aware of any large scale European population-based studies. The present study aimed to gain a better understanding of the incidence and outcome for this under-recognised clinicopathogical entity, by using the database of the Thames Cancer Registry for South East England to obtain new information on EPSCC. Registry datasets are potentially an efficient initial way of using existing information before undertaking a more specific and extensive clinical study of an uncommon cancer. Our objectives were to 1) compare the incidence and survival of EPSCC with that for SCLC (a related but more common neuroendocrine tumour), 2) determine the most common anatomical presenting sites for EPSCC, and 3) compare survival in EPSCC by disease stage and site of diagnosis. We hoped the study might stimulate further clinical and epidemiological studies investigating this disease.

## Methods

### Data

In the United Kingdom cancer registries record the occurrence of cancer in their residential populations. Registry data and analyses are held in publically funded data repositories and can be requested by researchers, clinicians, health care organisations, governments and the public. However this is subjected to ethical approval depending on the level of data requested. The Thames Cancer Registry (TCR) was originally established in 1960 as the South Thames Metropolitan Cancer Registry. This was a population-based registry covering the areas of South London, Kent, Surrey, Sussex and Wessex. In 1971–2 Wessex was excluded from the area and in 1985 the Thames Cancer Registry (TCR) was named and its area extended to cover North Thames areas including Essex, Hertfordshire and Bedfordshire. In 1996 Bedfordshire was excluded and by 2004 it covered an area of South East England including a population of 14 million people living in London, Essex, Hertfordshire, Kent, Surrey and Sussex. This study included cases from South London, Surrey, Sussex and Kent for the period 1970–1984 and from 1985–2004 it also included cases from North London, Hertfordshire and Essex. These well-defined areas have complete registration coverage for each time period and are recorded in TCR reports. In this area registration is now initiated by clinical and pathology information received from hospitals and by information about deaths provided by the National Health Service Central Register through the Office for National Statistics. Trained data collection officers collect information from the medical records on demographic, tumour details and on treatments received in the first six months after diagnosis. This includes the date of the first surgery, radiotherapy, chemotherapy and hormonal therapy. These data are added to a central database, quality assured and continually updated. Data on clinical performance status is routinely recorded in the UK in some clinical datasets for common cancers, but not yet in clinical practice or by the cancer registration system and was therefore not available for analysis.

During the Registry's history the data collection methods used (in line with those for other UK registries) have evolved from manual methods of recording information from medical records and copies of death certificates to an increasingly electronic transfer of pathology records from hospitals and death certificate data. Pathology reports have remained the main source of the diagnostic information since its inception. Only very occasionally will a pathological diagnosis be queried during the quality assurance process, and validation studies on the diagnoses made by pathologists are not routinely performed. The rate of misclassification of diagnoses is therefore unknown. Data on an unusually rare diagnosis like EPSCC are very likely to have been recorded from a pathology report and only very occasionally based on information from a death certificate. During some periods of the study the rate of registrations made in the TCR by death certificate only (DCO) was as high as 25%, leading some investigators to question the accuracy of survival figures calculated when these cases were excluded [12]. A recent study showed that the DCO rates declined substantially between the period 1990–4 and 1997–2001 across all sites [13]. The overall rate for 2004 data was 5% [14]. There is concern about comparing survival over area or time periods where DCO rates vary [13]. However, incidence and survival calculations for EPSCC are unlikely to be influenced unduly by varying DCO rates throughout the study because of the reliance on pathology reports.

### Selection of patients

The ICD-O morphology codes for small cell carcinoma (8041/3), oat cell carcinoma (8042/3), small cell carcinoma, fusiform cell (8043/3), small cell carcinoma, intermediate cell (8044/3), and combined small cell carcinoma (8045/3) were used to identify cases for the study. Using these definitions, data on a total of 29128 patients registered with small cell carcinoma between 1970 and 2004 were extracted from the database. Of these, 1618 (702 men and 916 women) were found to be diagnosed with EPSCC (5.8%). This group therefore excluded SCLC, secondaries and Merkel cell carcinomas of the skin. We also excluded data on patients diagnosed before 1970, as we were less certain about the reliability of diagnoses before this point. After this point we included all patients with this diagnosis and did not exclude any patients with missing data items.

### Anatomical site and disease stage

To determine the most common anatomical sites the data were grouped into the following main sites: head and neck, gastrointestinal tract, chest, breast, genitourinary tract, unknown primary of lymph nodes and others. Within these groups the data were further divided by individual site. The staging data received from hospitals by the Registry are not always complete, and a simplified scheme was therefore used for this study, taking into account all the information available. This defined three groups – 1) "limited disease" (a tumour confined to the primary site with or without local lymph node involvement), 2) "extensive disease" (any indication of disease spread beyond local boundaries) and 3) "unknown" (not recorded or not known). Detailed information on treatment protocols was not available, has changed during the study period and many of the site groups still include relatively small numbers of patients. We therefore decided not to present treatment analyses in this study.

### Statistical analyses

Age-standardised incidence rates for EPSCC and SCLC per 100,000 population were calculated using the European standard population for each five years of diagnosis to allow comparison with any future studies in the European region. These were plotted using a logarithmic scale to accommodate the large difference between them. Crude survival was measured from the time of diagnosis to the date of death or to the study censor date of 31/12/2004, and calculated using the Kaplan-Meier method. The crude overall survival of patients with EPSCC was compared to those with SCLC. Within the EPSCC group survival was then compared by stage of disease and by anatomical site. Due to the relatively small number of patients reaching five years for some sites in some of the analyses we only report the 3-year survival rates.

### Ethical Approval

Cancer registries in England carry out cancer surveillance using the data they collect under Section 60 of the Health and Social Care Act 2002. The study used an anonymised dataset and separate ethical approval was not required.

## Results

### The incidence and survival of EPSCC and SCLC

**Figure 1:**
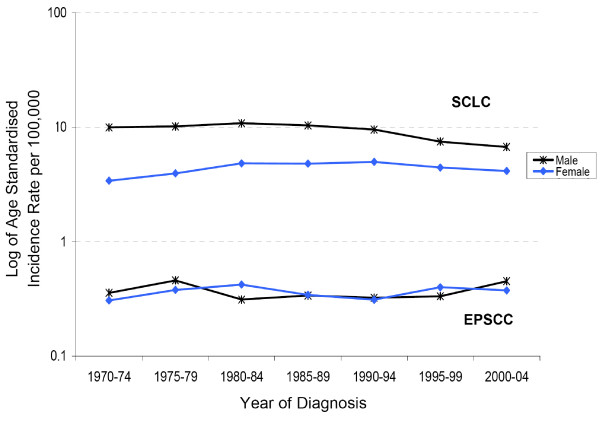
**Age-standardised incidence rates (ASR) per 100,000 for EPSCC and SCLC in South East England, 1970–2004**.

Figure 1 shows the age-standardised incidence rates for SCLC and EPSCC in South East England between 1970 and 2004. The incidence of SCLC remained higher in males than in females although it declined in males and increased in females during the study period. By 2000–2004 the incidence of SCLC was 6.72 per 100,000 in males and 4.15 per 100,000 in females. That for EPSCC was stable throughout the study period and was 0.45 per 100,000 during 2000–2004 in males and 0.37 in females. This represents a 15-fold difference in incidence between the two diseases.

**Figure 2:**
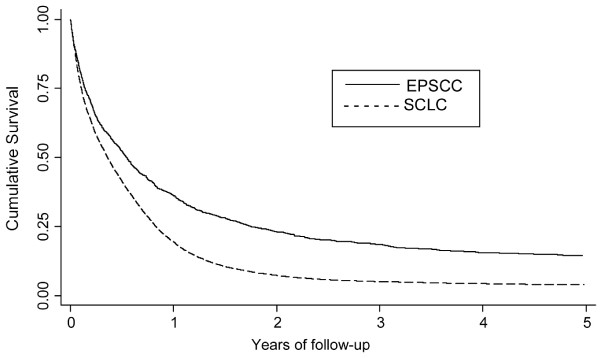
**Crude survival for patients with EPSCC and SCLC in South East England, 1970–2004**.

Figure 2 shows that patients with EPSCC had a better 3-year survival (19%) than those with SCLC (5%).

The median age at diagnosis for patients with EPSCC was 70 years (range, 0–85 years while that for SCLC was 65 years (range 20–85). The male: female ratio was 1:1.3 in EPSCC compared to 1.7:1 in SCLC. Table 1 shows that for EPSCC the most common presenting sites were gastrointestinal tract (33%), genitourinary tract (20%), head and neck (11%), and breast (10%). Oesophagus (18%, 293/1618) and breast (10%, 167/1618) were the two most common locations. As expected the rate of registrations made from death certificate only (DCO) is very generally low (1–3% for specific sites), suggesting that these pathological diagnoses and their registrations are nearly always made before death.

**Table 1:** Primary disease sites in patients with EPSCC in South East England, 1970–2004

Disease site	Number of Patients (%)	Location	(n)	Ratio (M:F)	Stage (n) [LD; ED; U]
Head and Neck	182 (11%)	Trachea	50	1:1.1	64; 30; 88
		Thyroid	41		
		Larynx	34		
		Oral Cavity	22		
		Sino-nasal	19		
		Pharynx	12		
		Others	2		
					
Gastrointestinal	532 (33%)	Oesophagus	293	1:1.3	181; 159; 192
		Stomach	92		
		Colorectal	60		
		Pancreas	55		
		Biliary Tract	15		
		Others	17		
					
Chest	115 (7%)	Heart, mediastinum and pleura	111	1.8:1	58; 14; 43
		Others	4		
					
Breast	167 (10%)			1:82	104; 13; 50
Genitourinary	305 (20%)	Prostate	90	1:1	109; 86; 110
		Bladder	79		
		Cervix	76		
		Ovary	44		
		Others	16		
					
Unknown Primary of Lymph Nodes	69 (4%)			1:1	0; 0; 69
Others	248 (15%)	Skin	11	1:1	16; 112; 120
		Others	237		
**EPSCC**	**1618 (100%)**			**1:1.3**	**532; 482; 604**

**SCLC**	**27510 (100%)**			**1.7:1**	**9284; 7651; 10575**

M: male; F: female; LD: limited disease; ED: extensive disease; U: unknown

**Figure 3:**
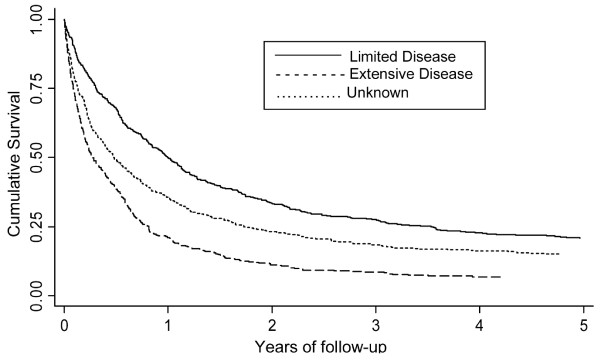
**Crude survival for patients with EPSCC by disease stage, South East England 1970–2004**.

Figure 3 shows that patients with limited disease at diagnosis had a better overall 3-year survival (28%) than those with extensive disease (9%) (median survival 1 year compared to 0.28).

**Figure 4:**
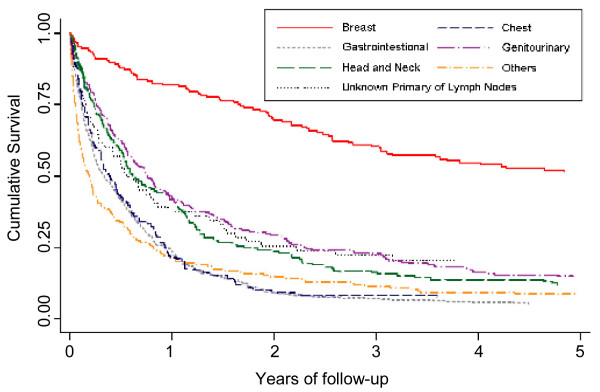
**Crude survival for patients with EPSCC by disease site, South East England 1970–2004**.

Figure 4 shows the survival after the diagnosis of EPSCC by disease site. The 3-year survival for the site groups were as follows: breast (60%), genitourinary system (23%), unknown primary of lymph nodes (22%), head and neck (16%), others (11%), chest (8%) and gastrointestinal (7%).

## Discussion

This study of 1618 patients registered with EPSCC in South East England is one of the largest studies so far published on this clinicopathological entity. Our findings confirm its rare nature in contrast to SCLC, which is a more widely studied and recognised cancer. There was a 15-fold difference in incidence between the two diseases. The incidence of EPSCC between 2000 and 2004 was 0.45 per 100,000 in males and 0.37 per 100, 000 in females and remained stable throughout the study period. This stable incidence and very little difference between the sexes in EPSCC suggest that its aetiological factors are more likely to be genetic and developmental rather than environmental, although this finding needs to be confirmed by other epidemiological studies. By contrast although the incidence of SCLC remained higher in males than females, it declined in males and increased in females reflecting different smoking patterns that are a known risk factor for the disease. Although we have compared EPSCC and SCLC to show the differences between them, they could be conceptualised as part of the same disease. The possibility that EPSCC may be a non-pulmonary cause of exposure to carcinogens related to smoking at lower doses has not often been considered. One Turkish study [15] suggested that fewer patients with EPSCC were smokers than with SCLC but it included only nine patients with EPSCC. A larger case control study should be undertaken to investigate this hypothesis more thoroughly.

In our study we found that the gastrointestinal (33%) and genitourinary (20%) tracts were the most common sites involved, whereas the oesophagus (18%) and breast (10%) were the two major organs affected. We also found a male-to-female ratio of 1:1.3, which differs from most other published studies, that find males are more commonly affected [6,16]. Previous studies have reported similar results to ours with the most common primary disease sites being oesophagus, breast, cervix, colon and rectum, head and neck and urinary bladder, although the most common site varies by study [3,6,7,16-18].

Our study found that the survival of EPSCC was higher (19%) than for SCLC (5%). Our analyses suggest that within EPSCC disease site is an important predictor of survival, with breast EPSCC standing alone as being associated with a better outcome compared to the rest of EPSCC as shown in Figure 4. Haider et al reported that patients with EPSCC of the breast had a good prognosis (median overall survival, 40.9 months) [6], and this trend was seen in patients with breast and genitourinary disease in other studies [6,7,16,19,20]. Generally these sites had a better survival than EPSCC of other sites. Similarly, in our study we found patients with gastrointestinal disease had the worst prognosis. Brenner et al also reported that the median survival of patients treated for EPSCC of the gastrointestinal tract ranged from 6 months to 12 months, and that very few patients survived in the longer term [21].

The overall 3-year survival of 30% for patients with limited disease and 10% for those with extensive disease is discouraging. The favourable outcome of 60% 3-year survival for patients with breast EPSCC compared to those with other sites may be attributed to earlier stage at presentation, since the majority of breast EPSCC in this study was limited stage disease. This may be due to the effect of the National Health Service (NHS) breast screening programme, introduced in 1988 [22], and the development of more effective treatments for breast cancer. Unfortunately data on screen-detected breast cancer is not available since the inception of the programme to test this hypothesis or to determine whether those detected through screening had a better prognosis. We do know that the incidence of breast cancer in England and Wales has increased during this period, in part due to the screening programme [23].

Despite being one of the largest series of patients so far there are several limitations to our study. The selection of cases was not based on a review of the pathology of cases. It is possible that some of the cases were misclassified secondaries from SCLC. This is most likely to be the case for EPSCC in the chest, and we have not analysed these cases separately. Conversely it is possible that cases of EPSCC were misdiagnosed by pathologists and not registered correctly, but the extent of any such misclassification is unknown. However, the steady rate of ascertainment of EPSCC in this population suggests that there was not a bias towards over-diagnosis due to increasing awareness of EPSCC during the years of study. Nonetheless, the overall survival for EPSCC of the breast and gastrointestinal tract, resemble other studies as mentioned above [6,7,16,17]. Exactly what factors contribute to the better or worse prognosis of various EPSCC sites could not be fully evaluated in our series. We cannot rule out the possibility that differences in natural history of these disease sites accounted for their better or worse survival. One problem with interpreting the literature is that there are many small studies of single disease sites. European studies [10,11] have generally included fewer than 25 patients. Meta-analyses of existing studies (where classification permits) might reveal more about the prognosis for disease at different sites.

Limited data are available in the literature describing the optimal management of patients with EPSCC. Because of the poor prognosis, combined treatment modalities have been increasingly used, with radiotherapy and/or surgery proposed for localised control and platinum-based chemotherapy proposed for systemic control [6]. However, there are no randomised controlled trials of treatment and it is difficult to draw conclusions about effective treatment from clinical series alone. This strongly suggests the need for further clinical studies.

At the time of the study, the Thames Cancer Registry database represents one quarter of the English cancer population. Further studies using the national UK database, the national cancer registration databases of other European countries and prospective case registers are recommended to confirm the results of this study. Such studies might include more patients with disease at individual sites, and, if augmented by case record review of patients with selected sites, could obtain more detailed information on treatment. The development of a co-ordinated research programme in these areas is clearly essential for better understanding of this under-recognised disease.

## Conclusion

EPSCC was identified in various anatomical sites, with the most common primary anatomical sites being the gastrointestinal and genitourinary tracts. The outcome for patients with EPSCC differed according to the primary disease site. Patients with EPSCC of the breast had the best prognosis, while EPSCC of the gastrointestinal tract had the worst survival. Consequently, the most sensitive predictor of survival was the presenting site and the extent of disease at diagnosis. Further studies using standardised diagnosis, prospective case registers for uncommon diseases and European cancer registries are needed to understand this disease.

## Competing interests

The authors declare that they have no competing interests.

## Authors' contributions

YNW conceived the idea for the study, helped design it, analysed the data and wrote the first draft of the paper. RHJ helped analyse the data, interpreted the findings and commented on the paper. VM helped to analyse the data, interpret the findings and commented on the paper. HM helped to design the study, interpret the findings and write the paper. EAD helped interpret the findings and revised all subsequent drafts of the paper. All authors have read and approved the final manuscript.

## Pre-publication history

The pre-publication history for this paper can be accessed here:

http://www.biomedcentral.com/1471-2407/9/209/prepub
